# Exploring the connectivity of dorsolateral prefrontal cortex and the modulatory impact of transcranial magnetic stimulation in adolescents with depression: a focus on pain-related cognitive processing

**DOI:** 10.1186/s12888-024-06321-x

**Published:** 2024-11-27

**Authors:** Dan Qiao, Yiran Li, Xiaoyu Zhang, Yujiao Wen, Xuemin Zhang, Lu Ma, Gaizhi Li, Chunxia Yang, Zhifen Liu

**Affiliations:** Department of Psychiatry, First Hospital of Shanxi Medical University, No. 85 Jiefang South Road, Taiyuan, 030001 China

**Keywords:** Adolescent depression, Pain, Cognition, Dorsolateral prefrontal cortex, Transcranial magnetic stimulation

## Abstract

**Background:**

Abnormal cognitive response to pain is consistently associated with deleterious outcomes among adolescents with depression. Highly relevant to both pain and cognition, dorsolateral prefrontal cortex (DLPFC) is important to understanding pain cognition. Our study aimed to characterize the circuit of DLPFC and the efficacy of transcranial magnetic stimulation (TMS) over DLPFC on pain cognition in adolescents with depression.

**Methods:**

Using neuroimaging data, we first compared functional connectivity (FC) of DLPFC between 60 adolescents with depression and 65 controls. The patients were then divided into add-on TMS group (*N* = 30) and Sertraline group (*N* = 30). Clinical outcome was determined using Pain Vigilance and Awareness Questionnaire (PVAQ) and Pain Catastrophizing Scale (PCS). Finally, we conducted regression analysis to assess the effect that FC of DLPFC contributes to in predicting clinical outcome.

**Results:**

FC analysis showed that compared to controls, patients displayed hyperconnection of left DLPFC - left triangular part of inferior frontal gyrus (IFG), which was significantly correlated with higher PCS total-, magnification- and helplessness-scores. Patients also showed hyperconnection of right DLPFC - right supramarginal gyrus (SMG), which was correlated with lower PCS total- and rumination- scores. After intervention, the add-on TMS group displayed significantly decreased score on PVAQ, PCS total and rumination. FC of left DLPFC - bilateral triangular part of IFG, - right SMG, as well as right DLPFC - left putamen, could predict the improvement of pain vigilance and magnification.

**Conclusion:**

Our results point to a key role of DLPFC acting as a connection linking cognitive control and pain processing in adolescents with depression.

**Trial registration:**

The study is registered in https://www.chictr.org.cn/ with a registration number ChiCTR2000039503 (date: 10.30.2020).

**Supplementary Information:**

The online version contains supplementary material available at 10.1186/s12888-024-06321-x.

## Introduction

Depression is the most common mental disorders among adolescents and presents considerable heterogeneity in its clinical manifestations [1]. Converging lines of evidence suggest that pain, the fifth vital sign, is a cardinal feature of adolescents with depression [2–5]. It is known that pain constitutes a multidimensional experience comprising components from the sensory, affective, and cognitive aspects. An important pain phenomenon is the notion of “pain paradox”, which can be described as an altered cognition to external pain stimuli [6]. Such phenomenon stressed the important impact of pain cognition, including pain catastrophizing and vigilance, in adolescents with depression.

Pain catastrophizing, characterized as an overly adverse examination to the pain experience, was associated with serious depressive symptom [7, 8]. And pain hypervigilance is a constant or amplified attention to pain, and further renders the focus on nociceptive input even more painful [9, 10]. Empirical research has shown that these pain cognitive components have additive adverse impacts on symptom manifestations, clinical prognosis, response to treatments in depression among adolescents [4, 11, 12]. Hence, to advance clinical management of depression in adolescence, it is imperative to conduct a comprehensive study of pain cognition, including mechanism and intervention [11, 13].

Despite the cognition of pain stems from processes that are highly distributed within the brain, mounting evidence suggests that a distributed overlapping between depression and pain processing across neuroimaging studies [14, 15]. Of these regions, the dorsolateral prefrontal cortex (DLPFC) might serve as a crucial node of networks that were involved in the processing of nociceptive information and pain regulation [13]. The DLPFC is typically linked to the regulation of top-down modulation and to the facilitation of proper behaviors [16]. Multiple strands of evidence back up the role of the DLPFC in suppressing pain perception and maintaining pain inhibition [13, 17]. One study where participants are provided with a sense of controllability regarding pain stimuli indicated that the DLPFC may be deeply engaged in the cognitive reaction towards pain [18]. However, it should be emphasized that functions cannot be ascribed to a single brain region separately. Concerning this matter, it is important to explore the circuit of DLPFC in cognitive components of pain among adolescents with depression.

In light of the convincing evidence that DLPFC function may reflect cognitive response to pain [19], it is possible that DLPFC could act as a therapeutic target. Actually, quite a few studies have lately shown that repetitive transcranial magnetic stimulation (TMS) of DLPFC could effectively manage sensory and emotional components of pain [20–23]. Specifically, repetitive TMS of the bilateral DLPFC could reduce pain intensity, either in acute or in chronic pain [13, 22]. And there was a correlation between greater left DLPFC activation and smaller differences in pain unpleasantness between depressed- and neutral- moods [24], offering support for its function of pain regulation in depression. However, it is currently unclear whether repetitive TMS can also improve their cognitive response to pain when targeting DLPFC.

Considering these points, we hypothesized that abnormal pain cognition would show a correlation with functional circuit of DLPFC, and TMS targeting DLPFC may have a modulatory effect on cognitive responses to pain among adolescents with depression. Here, we conducted a longitudinal study to (1) characterize the relevant functional circuit of DLPFC in cognitive response to pain among adolescents with depression, (2) explore the efficacy of TMS over DLPFC on negative cognition of pain, and (3) examine the predictive power of the neuro-biomarkers to explain clinical outcomes of pain catastrophizing and vigilance in adolescents with depression.

## Methods

### Participants

A total of 125 subjects aged 12–24 years were included in the current study. Among them, there were 60 drug-naïve adolescents who were experiencing their first episode of depression, and 65 healthy controls. The patients were enlisted from the Department of Psychiatry, the First Hospital of Shanxi Medical University, Taiyuan, China. As for the healthy controls, they were also recruited from Taiyuan, China, by the means of advertising in the local community. To identify whether or not the participants had any psychiatric diagnoses, each of the participants underwent independent evaluations by two trained psychiatrists using the Mini International Diagnostic Interview (M.I.N.I).

Patients were eligible if they were: (1) complied with the criteria for major depressive disorder according to the Diagnostic and Statistical Manual of Mental Disorders, Fifth Edition (DSM-5), and did not comply with the criteria for other disorders; (2) were experiencing their first episode; (3) had no prior history of receiving any treatment for depression, including pharmacological therapies, psychotherapies, and brain stimulation; (4) right-handedness; (5) ethnic Han. Healthy controls had no history of any psychiatric diagnosis. Participants were excluded if (1) were experiencing severe or unstable physical diseases or nervous system diseases; (2) with the presence of psychotic symptoms; (3) contraindications to magnetic resonance imaging (MRI) scans; (4) individual or family history of epileptic seizures.

All eligible subjects were explained that the aim of the study was to explore the mechanism of pain cognition and further find out whether receiving TMS might help adolescents with depression to relief altered cognitive responses to pain. Patients were also notified that they had a 1/2 chance of getting TMS intervention for free as a supplementary support. It should be emphasized that this intervention was not meant to replace or restrict their use of antidepressants. The study received approval from the Ethics Committee of the First Hospital of Shanxi Medical University, where the approval number was K062. In the meantime, all the participants and their guardians signed the informed consent.

### Measures

#### Clinical assessment

Demographic data were self-reported at baseline by all participants, including sex, age, and educational level, and the symptom questionnaire were conducted at baseline and post-intervention. The primary efficacy outcomes were the Pain Catastrophizing Scale (PCS) [25] and Pain Vigilance and Awareness Questionnaire (PVAQ) [26, 27], both well-established and self-reported measure of pain cognition. PCS, consisting of 13 items within 3 subscales, measured the subjective appraisals of helplessness, magnification, and rumination during the experience of pain. Each item was scored based on a 5-point Likert scale that ranging from 0 (not at all) to 4 (extremely). Subsequently, the scores were combined together to generate a composite PCS score, and a higher score implies a greater level of pain catastrophizing [28]. And PVAQ was conducted to assess various aspects of pain-related attention, such as awareness, vigilance, preoccupation, and observation of pain. Comprising 16 items in total, each item was rated on a 6-point Likert scale that spanned from 0 (never) to 5 (always) [29].

The secondary efficacy outcomes were the 24 item Hamilton Depression Rating Scale (HAMD-24) and Hamilton Anxiety Rating Scale. HAMD-24 and HAMA were both interviewer-rated scale, and all assessors underwent training given by one expert in psychological assessment. Items of HAMD-24 are scored on a 5-point Likert scale (0–4), with higher scores indicating more depressive symptoms [30], while HAMA consists of 14 items on a 5-point Likert scale (0–4), with higher scores indicating more anxiety symptoms [31].

#### Image acquisition and processing

##### MRI Acquisition

The structural and functional MRI data were collected at baseline, using a 3.0T MR system (Siemens Skyra) with a 20-channel birdcage head coil. It was mandatory for the participants to keep their eyes closed while staying awake and relaxed during the entire scanning. Rubber earplugs and foam pads were utilized to minimize the head motion and noise interference. For each participant, an MPRAGE Sagittal sequence was used to acquire a T1-weighted structural image. The parameters for this sequence were as follows: echo time (TE) = 3.97 ms, repetition time (TR) = 1900 ms, flip angle (FA) = 8°, acquisition matrix = 192 × 192, 192 slices, slice thickness = 1 mm, with no gap. And a functional data was obtained using an echo-planar imaging sequence. The details of its parameters were: TE = 2620/30 ms, TR = 2620 ms, FA = 90°, field of view = 192 × 192 mm, acquisition matrix = 64 × 64, slice thickness = 3 mm, with no gap, 47 slices, 220 volumes.

##### Resting-state functional MRI Data Preprocessing

The preprocessing of the resting-state functional MRI data was conducted by Data Processing and Analysis for Brain Imaging software (DPABI; DPABI_V8.1_240101, http://rfmri.org/dpabi). First, the initial 10 volumes, which were meant to enable the participants get accustomed to the scanning environment, were removed. Next, slice timing as well as head-motion correction was implemented on all of the remaining images. Then, the images were co-registered to the T1 anatomical images, which were further transformed into the Montreal Neurological Institute (MNI) space with the resampled voxel size of 3 × 3 × 3 mm^3^ voxels. Afterward, the functional images were smoothed by applying a Gaussian kernel with a full width at half maximum (FWHM) of 4 mm to cut reduce noise. Subsequently, linear detrending and temporal band-pass filtering (within the frequency range of 0.01–0.08 Hz) were carried out. Finally, Friston-24 motion parameters, cerebrospinal fluid, and white matter signals were regressed out from the time series. It is noteworthy that subjects with a maximum head movement that was greater than 2.0 mm in displacement or more than 2.0° in rotation were not considered in the final analysis. And in order to evaluate the frame-wise head motion confound, we made a comparison between the mean framewise displacement (FD) of patients with depression and that of the controls.

##### FC calculation and analysis

By extracting the residual Blood Oxygenation Level Dependent (BOLD) signal time course from regions of interest (ROI), we generated the whole-brain seed-to-voxel correlation maps. The seed regions, which were 5 mm in diameter, were created according to the peak coordinates [32]. Specifically, for the left DLPFC, the peak coordinates were [x = − 43, y = 22, z = 34], while for the right DLPFC, they were [x = 43, y = 22, z = 34]. Then, we extracted the mean BOLD time course from each seed and computed the Pearson’s correlation coefficients of this mean BOLD time course with the time course of all other voxels within the brain. Finally, the correlation coefficients were transformed into Z values by Fisher’s *r*-to-*z* transformation.

To compare FC of DLPFC and identify abnormalities between patients and controls, two-sample *t*-tests were performed with age, gender, years of education and mean FD included as covariates. The group differences were considered significant for voxel *P* value < 0.001 with cluster *P* value < 0.05 (Gaussian random field corrected). In order to further explore the correlations existing between pain cognition and neural patterns among adolescents suffering from depression, we conducted Pearson’s partial correlation analyses (two-tailed) between FC values showing significant group differences and the score of PVAQ, PCS, and PCS-subscale, while taking age, gender, and years of education into account as controlled variables. Statistically, a *P* value less than.05 was considered to be significant.

### Intervention

All eligible patients were randomly allocated to either the Add-on TMS group or the Sertraline group at a 1:1 ratio. And the symptom evaluators were blinded to the treatment group.

(1) The Sertraline group: treatment was carried out using Sertraline. Its daily dose varied between 50 and 200 mg, while the initial daily dose was from 25 to 50 mg.

(2) The Add-on TMS group: based on medication, patients received 5 sessions TMS intervention per week for 2 weeks.

Robot-aided navigation: A robotic device (Yiruide Co., Ltd., Wuhan, China) was used to locate the DLPFC. Such device aims to manipulate and reposition the magnetic coil over the patient’s head, adjusting to their movements to maintain a consistent stimulation site and ensuring the coil is correctly oriented. The orientation including 5 steps: (1) during the first treatment, the patient’s head circumference, the distance between the ear tips, and the distance between the eyebrows and the occipital bone are measured to establish the data of the personal head model; (2) adjusting the position of the bed until the camera recognizes patient’s face and the screen displays the optimal position of the six points; (3) selecting the target point that the patient needs to stimulate, and save the target point; (4) verifying the target point: ensure that the deviation range is within 5 mm, then the target point creation is complete.

TMS intervention: Given the clinically meaningful benefits of bilateral TMS in both pain and depression [20, 33, 34], we chose bilateral repetitive TMS protocol in our study. Magnetic pulses were generated by NS 5000 magnetic stimulators (Yiruide Co., Ltd., Wuhan, China). Stimulation was delivered at an intensity of 80% of the patient’s resting motor threshold (RMT) with high-frequency (HF) repetitive TMS on the left DLPFC (10 Hz, 30 trains, 5 s trains, 15 s intertrain interval, 1500 pulses per session) and low-frequency (LF) repetitive TMS on the right DLPFC (1 Hz, 6 trains, 100 s trains, 30 s intertrain-interval, 600 pulses per session). The RMT needs to be re-measured after each adjustment of drug dose.

### Statistical analysis

Descriptive statistics were performed on the demographic and clinical characteristics in each group, including means and standard deviations. Two-sample *t*-tests and Chi-square tests were used to assess demographic and clinical variable differences between the patients and controls and between the Add-on TMS group and the Sertraline group.

To explore the improvements in clinical symptoms, the paired *t*-tests were utilized to detect the changes of dependent variables (PVAQ score, PCS score, PCS-subscale score, HAMD-24 score, and HAMA score) from the pre- to post-intervention in each group (the Add-on TMS group and the Sertraline group), whereas the two-sample *t*-tests were used to detect group differences between the Add-on TMS group and the Sertraline group at each timepoint. And to explore the connection between the improvement of depressive symptoms and pain cognition, we conducted additional statistical analyses relying on binary classification of depressive symptoms trajectory, i.e. responders vs. non-responders. Patients in add-on TMS group were further divided into responders or non-responders after intervention. Specifically, a subject was considered as a responder if the total score HAMD-24 after intervention had decreased by 50% or more compared to the baseline score. We then compared the difference in demographic variables, depressive and anxiety symptoms (HAMD-24 and HAMA), and pain cognition (PVAQ score, PCS score, and PCS-subscale) between responders and non-responders by using two-sample *t*-tests, and changes from baseline to post-intervention in pain cognition were assessed both in responders and non-responders by using paired *t*-tests.

In order to assess the potential of neuro-biomarker serves as predictor of clinical outcomes of pain cognition, we performed hierarchical regression analyses with the dependent variables as reduction in PVAQ score, PCS total, and subscale score in the add-on TMS group. At first, the baseline covariates for models were examined through scatter plots. Those that demonstrated significant bivariate correlations with outcome measures were then selected. Subsequently, the independent variables were inserted into the hierarchical model following this order: (1) control variables (age, gender, and education level); (2) clinical confounders (baseline HAMD-24, HAMA, PVAQ, and PCS score); (3) FC showing significant differences between patients and controls. The outcome variables were reported in differences (*β*) along with 95% confidence intervals (CI). Statistically, a *P* value less than.05 was considered to be significant. Analyses were conducted using IBM SPSS Statistics for Windows.

## Results

### Demographic and clinical characteristics between patients with depression and controls

Table 1 shows the demographic and clinical characteristics of all subjects. There were no significant differences between the two groups in terms of age, gender, years of education, or PVAQ scores (all *P* > .05). The score of HAMA, HAMD-24, PCS, PCS-rumination, PCS-helplessness, and PCS-magnification in the depression group were higher than in the controls (all *P* < .05). Besides, no subjects were excluded after quality control of MRI data, and there were no significant differences between the two groups in terms of the mean FD (Mean ± SD, Patients with Depression vs. Controls: 0.06 ± 0.03 vs. 0.06 ± 0.02, *t* = 1.065, *P* = .289).

**Table 1:** Demographics and clinical characteristics for subjects included in this study

	Patients with depression (*N* = 60)	Controls (*N* = 65)	t or χ²	*P* value
Age, mean (SD)	16.40 (2.125)	16.58 (1.878)	−0.516	0.607
Gender (male/female)	13/47	14/51	< 0.001	0.986
Education level, mean (SD)	10.25 (2.440)	10.09 (2.350)	0.368	0.714
HAMD-24 score, mean (SD)	22.47 (5.629)	1.48 (2.532)	27.233	< 0.001
HAMA score, mean (SD)	15.93 (5.079)	0.91 (1.852)	22.293	< 0.001
PVAQ score, mean (SD)	36.20 (11.865)	32.95 (12.146)	1.510	0.134
PCS total score, mean (SD)	24.55 (13.720)	12.60 (10.913)	5.409	< 0.001
PCS-rumination sub-score, mean (SD)	7.27 (4.411)	4.38 (3.860)	3.895	< 0.001
PCS-helplessness sub-score, mean (SD)	11.43 (6.828)	5.38 (5.092)	5.642	< 0.001
PCS-magnification sub-score, mean (SD)	5.85 (3.328)	2.83 (2.673)	5.612	< 0.001

*HAMD-24* 24 item Hamilton Depression Rating Scale, *HAMA* Hamilton Anxiety Rating Scale *PVAQ* Pain Catastrophizing Scale, *PCS* Pain Vigilance and Awareness Questionnaire

### FC differences between patients with depression and controls

**Fig. 1:**
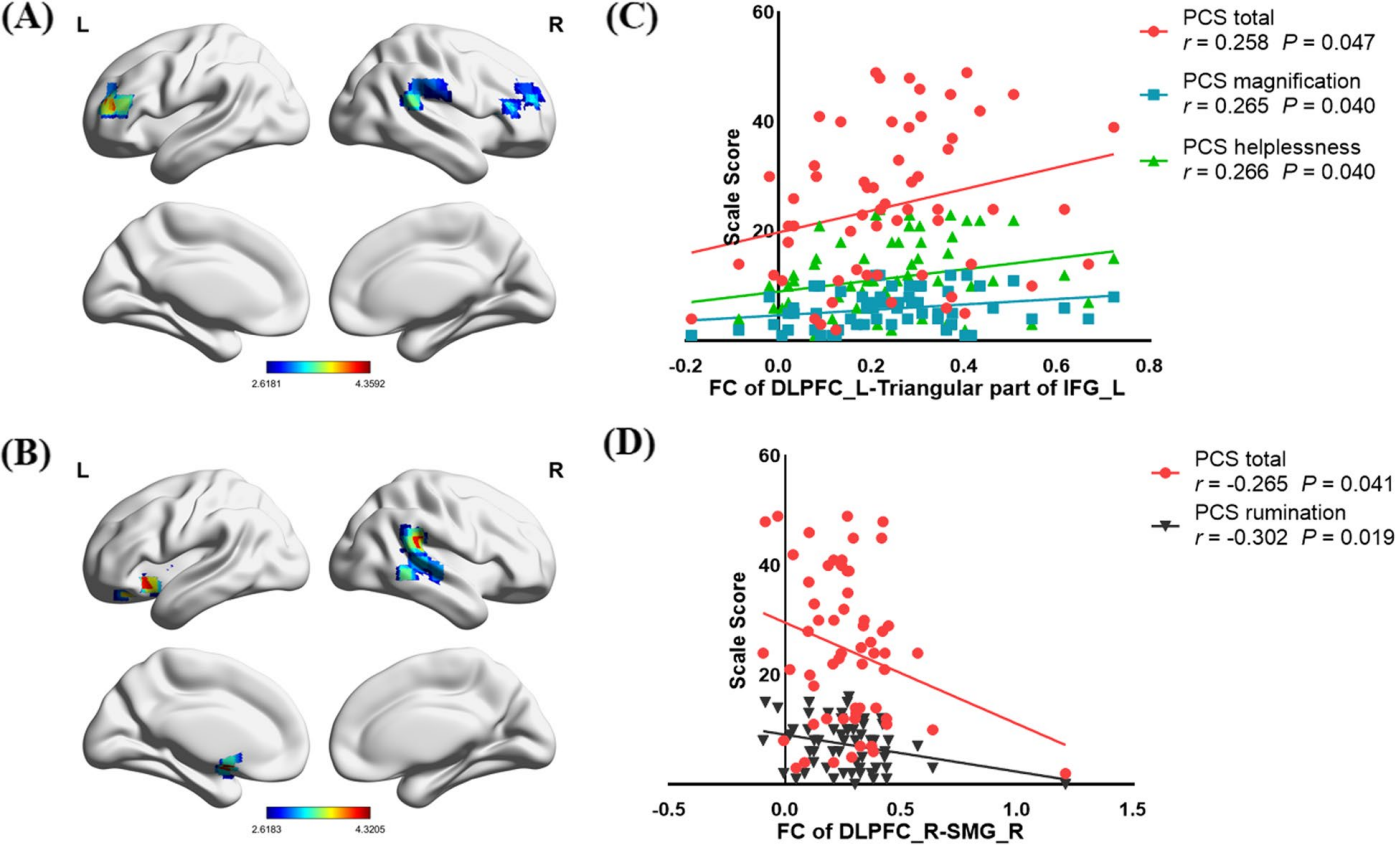
Significant differences in functional connectivity (FC) of left (A) dorsolateral prefrontal cortex (DLPFC) and right DLPFC (B) between adolescents with depression and controls. The color bars indicate the *t*-value based on two-sample *t* tests. Significant at *P* < .001, corrected by Gaussian random field (GRF) correction. Correlation between FC of left (C) DLPFC and (D) DLPFC and pain catastrophizing dimensions in adolescents with depression. PCS, Pain Vigilance and Awareness Questionnaire. IFG, inferior frontal gyrus. SMG, supramarginal gyrus

Age-, gender-, years of education-, and mean FD-adjusted two-sample *t*-tests revealed that compared with controls, adolescents with depression exhibited increased FC between the left DLPFC and the bilateral triangular part of inferior frontal gyrus (IFG) and right supramarginal gyrus (SMG), also with increased FC between right DLPFC and left putamen and right SMG (Fig. 1; Table 2).

**Table 2:** Brain regions showing significant FC differences between patients with depression and healthy control

Brain Region	Cluster size	MNI coordinates			t value
		X	Y	Z	
ROI: DLPFC_L					
Triangular part of IFG_R	165	42	24	12	3.870
Triangular part of IFG_L	164	−33	30	15	4.360
SMG_R	195	69	−30	18	4.078
ROI: DLPFC_R					
Putamen_L	146	−9	6	−9	4.321
SMG_R	204	54	−36	21	4.066

*DLPFC* dorsolateral prefrontal cortex, *IFG* inferior frontal gyrus, *SMG* supramarginal gyrus

### Correlation analysis between FC and pain cognition

FC between left DLPFC and left triangular part of IFG showed a positive correlation with the total of PCS score (*r* = .258, *P* = .047), as well as the magnification (*r* = .265, *P* = .040) and helplessness subscore (*r* = .266, *P* = .040). Additionally, FC between right DLPFC and right SMG were negatively correlated with the total score (*r* = -.265, *P* = .041) and rumination subsore (*r* = -.302, *P* = .019) of PCS (Fig. 1).

### Demographic characteristics between add-on TMS group and sertraline group

No significant differences were observed between the two groups in terms of age (Mean ± SD, Add-on TMS vs. Sertraline: 15.83 ± 2.001 vs. 16.87 ± 2.193, *t* = −1.906, *P* = .062), gender (male/female, Add-on TMS vs. Sertraline: 5/25 vs. 8/22, *χ²* = 0.884, *P* = .347), or educational level (Mean ± SD, Add-on TMS vs. Sertraline: 9.80 ± 2.295 vs. 10.80 ± 2.52, *t* = −1.065, *P* = .114).

### Changes in clinical symptoms after the intervention between add-on TMS group and sertraline group

Table 3 showed that changes in the clinical symptoms after the intervention between groups. No significant differences in clinical variables were observed between the two groups at baseline (all *P* > .05). For depressive and anxiety symptoms, both Add-on TMS group and Sertraline group showed significant decreased scores in HAMD-24 (*t* = 7.645, *P* < .001) and HAMA (*t* = 7.755, *P* < .001). And in terms of pain cognition, compared with baseline, the PVAQ score (*t* = 3.864, *P* < .001), PCS total score (*t* = 5.164, *P* < .001), PCS-rumination score (*t* = 4.460, *P* < .001), PCS-helplessness score (*t* = 4.418, *P* < .001), and PCS-magnification score (*t* = 4.368, *P* < .001) were all decreased in Add-on TMS group, whereas only the PCS total score (*t* = 3.427, *P* = .002), and PCS-helplessness sub-score (*t* = 4.313, *P* < .001) significantly decreased after the intervention in the Sertraline group.

**Table 3:** Changes in clinical symptoms after the intervention between groups

Measure	Add-on TMS group (*N* = 30)	Sertraline group (*N* = 30)	Difference(95%CI)	*P* value^a^	Cohen’s d
**PVAQscore**, mean (SD)					
Baseline	35.63 (11.961)	36.77 (11.944)	−1.13 (−7.311, 5.044)	0.715	−0.10
Post-intervention	26.00 (12.817)	35.27 (11.750)	−9.267 (−15.621, −2.912)	0.005	−0.75
Difference(95%CI)	9.633 (4.535, 14.732)	1.500 (−2.987, 5.987)	N/A	N/A	N/A
* P* value^b^	< 0.001	0.500	N/A	N/A	N/A
Cohen’s *d*	0.78	0.13	N/A	N/A	N/A
**PCS total score**, mean (SD)					
Baseline	23.53 (11.741)	25.57 (15.589)	−2.033 (−9.166, 5.099)	0.570	−0.15
Post-intervention	15.90 (10.430)	22.43 (13.132)	−6.533 (−12.662, −0.404)	0.037	−0.55
Difference(95%CI)	7.633(4.610, 10.657)	3.133(1.263, 5.004)	N/A	N/A	N/A
* P* value	< 0.001	0.002	N/A	N/A	N/A
Cohen’s *d*	0.69	0.22	N/A	N/A	N/A
**PCS-rumination sub-score**, mean (SD)					
Baseline	6.87 (3.884)	7.67 (4.915)	−0.800 (−3.089, 1.489)	0.487	−0.18
Post-intervention	4.50 (3.160)	7.40 (4.889)	−2.900 (−5.027, −0.773)	0.008	−0.70
Difference(95%CI)	2.367 (1.281, 3.452)	0.267 (−0.648, 1.181)	N/A	N/A	N/A
* P* value	< 0.001	0.555	N/A	N/A	N/A
Cohen’s *d*	0.67	0.06	N/A	N/A	N/A
**PCS-helplessness sub-score**, mean (SD)					
Baseline	10.90 (5.985)	11.97 (7.645)	−1.067 (−4.615, 2.482)	0.550	−0.16
Post-intervention	7.30 (5.682)	9.63 (6.088)	−2.333 (−5.377, 0.710)	0.130	−0.40
Difference(95%CI)	3.600 (1.825, 5.375)	2.333 (1.227, 3.440)	N/A	N/A	N/A
* P* value	< 0.001	< 0.001	N/A	N/A	N/A
Cohen’s *d*	0.62	0.34	N/A	N/A	N/A
**PCS-magnification sub-score**, mean (SD)					
Baseline	5.77 (2.775)	5.93(3.850)	−0.167 (−1.901, 1.568)	0.848	−0.05
Post-intervention	4.10 (2.808)	5.40(3.856)	−1.300 (−3.043, 0.443)	0.141	−0.39
Difference(95%CI)	1.667 (0.886, 2.447)	0.533 (−0.348, 1.414)	N/A	N/A	N/A
* P* value	< 0.001	0.226	N/A	N/A	N/A
Cohen’s *d*	0.60	0.14	N/A	N/A	N/A
HAMD-24 **total score**, mean (SD)					
Baseline	23.40 (5.998)	21.57 (5.177)	1.833 (−1.062, 4.729)	0.210	0.33
Post-intervention	15.20 (8.475)	16.50 (7.825)	−1.300 (−5.515, 2.915)	0.539	−0.15
Difference(95%CI)	8.200 (6.006, 10.394)	5.067 (2.325, 7.809)	N/A	N/A	N/A
* P* value	< 0.001	< 0.001	N/A	N/A	N/A
Cohen’s *d*	1.12	0.76	N/A	N/A	N/A
HAMA **total score**, mean (SD)					
Baseline	16.40 (4.847)	15.47 (5.329)	0.933 (−1.699, 3.566)	0.481	0.18
Post-intervention	9.87 (5.476)	11.37 (5.530)	−1.500 (−4.344, 1.344)	0.295	−0.27
Difference(95%CI)	6.533 (4.810, 8.256)	4.100 (1.900, 6.300)	N/A	N/A	N/A
* P* value	< 0.001	< 0.001	N/A	N/A	N/A
Cohen’s *d*	1.26	0.76	N/A	N/A	N/A

^a^*P* value of independent-samples *t* test. ^b^*P* value of paired *t* test. *TMS* Transcranial Magnetic Stimulation, *PVAQ* Pain Vigilance and Awareness Questionnaire, *PCS* Pain Catastrophizing Scale, *HAMD-24* 24 item Hamilton Depression Rating Scale, *HAMA* Hamilton Anxiety Rating Scale, *N/A* not applicable

### Demographic and clinical characteristics between responders and non-responders in add-on TMS group

Table S1 shows the demographic and clinical characteristics of responders and non-responders in Add-on TMS group. There were no significant differences between responders and non-responders in terms of age, gender, educational level, or HAMD-24 scores (all *P* > .05). The HAMA score in the responders were lower than in the non-responders (*t* = −2.917, *P* = .007).

### Changes in pain cognition after the intervention between responders and non-responders in add-on TMS group

Table S2 showed that changes in the pain cognition after the intervention between responders and non-responders in Add-on TMS group. At baseline, the scores of PCS and PCS-magnification were both lower in responders compared to those in non-responders (all *P* < .05). Compared with baseline, the PCS total score, PCS-rumination score, PCS-helplessness score, and PCS-magnification score were all decreased in both responders and non-responders after intervention (all *P* < .05), whereas only the responders showed a significant reduction in PVAQ score (*t* = 4.027, *P* = .002).

### Hierarchical regression analyses predicting the change of pain cognition in add-on TMS group

The final multivariable regression model predicting the change of PVAQ score after add-on TMS was significant and explained 68.8% variance of PVAQ score reduction (*F* = 3.126, *P* = .016). Notably, the predictive effects of part FC of DLPFC on the change of PVAQ score were significant, including the FC of left DLPFC and right SMG (*β* = 65.547, *P* = .037) and FC of right DLPFC and left putamen (*β* = − 80.525, *P* = .005). Besides, the final multivariable regression model predicting the change of PCS-magnification score was also significant and explained 56.6% variance of PCS-magnification score reduction (F = 2.896, *P* = .023). Of this model, the predictive effects of part FC of DLPFC on the change of PCS-magnification score were significant, including the FC of left DLPFC and bilateral triangular part of IFG (left: *β* = −13.381, *P* = .017; right: *β* = 14.761, *P* = .027, Table 4).

**Table 4:** Hierarchical regression analyses predicting the change of pain cognition in add-on TMS group

step	Variables	PVAQ score			PCS total score			PCS-rumination sub-score			PCS-helplessness sub-score			PCS-magnification sub-score		
		*β*	95%CI	*P* value	*β*	95%CI	*P* value	*β*	95%CI	*P* value	*β*	95%CI	*P* value	*β*	95%CI	*P* value
1	age	−0.747	−4.177 to 2.683	0.658	0.222	−2.009 to 2.453	0.840	−0.187	−0.982 to 0.609	0.634	0.223	−1.080 to 1.526	0.728	0.186	−0.386 to 0.757	0.510
	gender	−10.932	−27.769 to 5.905	0.194	3.827	−7.124 to 14.779	0.479	1.225	−2.683 to 5.132	0.525	2.143	−4.253 to 8.538	0.497	0.460	−2.345 to 3.266	0.739
	education level	−1.316	−4.325 to 1.694	0.377	−0.075	−2.032 to 1.883	0.938	−0.086	−0.785 to 0.612	0.801	0.016	−1.127 to 1.159	0.977	−0.004	−0.506 to 0.497	0.986
2	age	−0.909	−4.255 to 2.437	0.579	−0.423	−2.633 to 1.786	0.695	−0.560	−1.205 to 0.084	0.085	0.015	−1.329 to 1.358	0.982	0.122	−0.520 to 0.764	0.698
	gender	−8.470	−27.089 to 10.148	0.356	−2.176	−14.468 to 10.116	0.717	−1.621	−5.207 to 1.965	0.358	−1.079	−8.553 to 6.395	0.768	0.524	−3.050 to 4.097	0.764
	education level	−1.120	−3.946 to 1.706	0.420	−0.008	−1.874 to 1.858	0.993	−0.042	−0.586 to 0.502	0.874	0.023	−1.111 to 1.158	0.966	0.011	−0.532 to 0.553	0.968
	HAMD-24 score	0.195	−0.856 to 1.245	0.704	0.130	−0.564 to 0.823	0.702	−0.003	−0.206 to 0.199	0.973	0.183	−0.238 to 0.605	0.377	−0.050	−0.252 to 0.151	0.610
	HAMA score	0.476	−0.751 to 1.702	0.430	0.046	−0.763 to 0.856	0.907	−0.088	−0.324 to 0.149	0.450	0.080	−0.412 to 0.573	0.738	0.053	−0.182 to 0.289	0.643
	PVAQ score	−0.401	−0.909 to 0.107	0.116	0.107	−0.228 to 0.443	0.513	0.062	−0.036 to 0.160	0.204	0.064	−0.140 to 0.268	0.524	−0.018	−0.115 to 0.079	0.705
	PCS total score	−0.199	−0.775 to 0.378	0.482	−0.449	−0.830 to −0.068	0.023	−0.185	−0.296 to −0.074	0.002	−0.241	−0.472 to −0.009	0.042	−0.023	−0.134 to 0.087	0.667
3	age	−2.012	−5.503 to 1.480	0.241	−0.753	−3.361 to 1.856	0.551	−0.763	−1.624 to 0.097	0.079	−0.076	−1.731 to 1.580	0.924	0.086	−0.620 to 0.792	0.800
	gender	−14.151	−32.615 to 4.313	0.124	1.512	−12.285 to 15.308	0.820	−1.409	−5.960 to 3.142	0.522	1.136	−7.618 to 9.890	0.788	1.785	−1.950 to 5.519	0.32
	education level	−0.369	−3.245 to 2.506	0.790	0.101	−2.048 to 2.249	0.923	0.101	−0.608 to 0.810	0.767	−0.039	−1.403 to 1.324	0.952	0.039	−0.543 to 0.620	0.890
	HAMD-24 score	0.322	−0.572 to 1.215	0.458	0.222	−0.445 to 0.890	0.492	0.012	−0.208 to 0.232	0.909	0.234	−0.189 to 0.658	0.260	−0.024	−0.205 to 0.157	0.782
	HAMA score	0.526	−0.629 to 1.681	0.350	−0.055	−0.918 to 0.808	0.894	−0.134	−0.418 to 0.151	0.336	0.050	−0.497 to 0.598	0.849	0.028	−0.205 to 0.262	0.802
	PVAQ score	−0.577	−1.092 to −0.061	0.030	−0.123	−0.508 to 0.262	0.509	0.032	−0.096 to 0.159	0.607	−0.053	−0.297 to 0.192	0.654	−0.102	−0.206 to 0.002	0.055
	PCS total score	−0.214	−0.751 to 0.324	0.413	−0.323	−0.724 to 0.079	0.108	−0.178	−0.310 to −0.045	0.012	−0.177	−0.432 to 0.078	0.161	0.032	−0.077 to 0.140	0.546
	FC of DLPFC_L-Triangular part of IFG_L	−32.874	−85.691 to 19.943	0.207	−33.169	−72.635 to 6.297	0.094	−3.375	−16.394 to 9.644	0.592	−16.413	−41.453 to 8.627	0.185	−13.381	−24.063 to −2.699	0.017
	FC of DLPFC_L-Triangular part of IFG_R	−6.467	−69.870 to 56.936	0.832	33.674	−13.703 to 81.050	0.152	3.919	−11.709 to 19.547	0.604	14.994	−15.065 to 45.053	0.307	14.761	1.938 to 27.584	0.027
	FC of DLPFC_L-SMG_R	65.547	4.341 to 126.752	0.037	−2.170	−47.905 to 43.564	0.921	1.386	−13.700 to 16.473	0.849	−1.416	−30.433 to 27.601	0.919	−2.141	−14.519 to 10.238	0.720
	FC of DLPFC_R-Putamen_L	−80.525	−133.496 to −27.553	0.005	−23.075	−62.657 to 16.506	0.235	−2.914	−15.971 to 10.143	0.644	−15.321	−40.434 to 9.793	0.215	−4.841	−15.554 to 5.872	0.354
	FC of DLPFC_R-SMG_R	−1.362	−42.329 to 39.605	0.945	−7.936	−38.547 to 22.676	0.592	−4.768	−14.866 to 5.329	0.333	−0.973	−20.395 to 18.449	0.917	−2.195	−10.480 to 6.091	0.584

*P* value < 0.05 was considered significant, represented with the corresponding 95% CI. *β*: the unstandardized regression coefficient

*HAMD*-24 24 item of Hamilton Depression Scale, *HAMA* Hamilton Anxiety Scale, *PVAQ* Pain Vigilance and Awareness Questionnaire, *PCS* Pain Catastrophizing Scale, *DLPFC* dorsolateral prefrontal cortex, *IFG* inferior frontal gyrus, *SMG* supramarginal gyrus

## Discussion

The current study investigated the role of DLPFC circuit in cognitive reaction to pain among adolescents with depression, with a focus on the efficacy of TMS as well as the predictive biomarker of pain cognition change. The main findings were: (1) compared with controls, adolescents with depression exhibit heightened pain catastrophizing; (2) adolescents diagnosed with depression existed heightened FC between DLPFC and SMG, putamen, and the triangular part of IFG. Part of these FC showed a significant correlation with pain cognition; (3) the add-on TMS group showed a pronounced alleviation in pain catastrophizing after intervention, regardless of whether depressive symptoms improved; (4) certain FC patterns of the DLPFC may serve as predictive biomarkers for the treatment outcome, offering a potential prognostic tool for clinical applications.

Although we didn’t identify the differences in pain vigilance between patients and control, adolescents with depression showed a higher level of pain catastrophizing, which manifest in various ways, including rumination, magnification, and helplessness. In line with previous studies, patients with depression tend to have a more negative perspective on the pain, which exacerbated their pain perception [35]. Such ideas also could contribute to a cycle of avoidance and withdrawal from academic or social activities, further leading to a deterioration in overall quality of life.

Among the findings, FC of DLPFC and triangular part of IFG showed a positive correlation with pain catastrophizing level, especially the magnification and helplessness of pain. Triangular part of IFG was a key node of ventral attention network, which is a “bottom-up” network, long recognized for its role in the reorienting of external attention to salient cues [36]. In this context, our results indicated an increased attention towards painful stimulus and salience detection may lead to the fear of pain. In addition, we also found the FC between DLPFC and SMG was negatively related to pain catastrophizing level, especially the rumination of pain. The SMG is thought to be essential for self-referencing, internal attention, and overcoming emotional egocentric bias [37]. Applying an altered self-referential projection framework in pain experience can result in self-oriented prejudiced appraisals. Combining the critical role of DLPFC in cognition control, the hypo-connection between these two regions may indicate an inadequate ability to control internal attentional bias to the disastrous consequences, which potentiates negative cognitive components of the pain experience [38].

Given the burden of negative pain cognition in adolescents with depression, and in light of crucial role of DLPFC in cognition control, we further explored the efficacy of TMS targeting DLPFC on negative pain cognition. Prior studies have shown that TMS of DLPFC can effectively regulate both acute and chronic pain perception, which could be mediated by effects on cognitive aspect of the pain experience [13]. The present results confirm that TMS targeting DLPFC does ameliorate negative pain cognition in adolescents with depression. Interestingly, we also found whether depressive symptoms responded or not, patients showed improvement in pain catastrophizing after TMS stimulation. Likewise, several studies indicated that the improvement of pain perception were not strongly associated with the amelioration of depression [39, 40]. Therefore, the clinical management of adolescents with depression might be remarkably strengthened when attention is paid to both pain cognition and depressive symptom.

Notably, we found hyperconnectivity of left DLPFC and left triangular part of IFG, as well as hypoconnectivity of left DLPFC and right triangular part of IFG, were associated with poor treatment outcomes of pain magnification. Previous studies have demonstrated the difference in bilateral triangular part of IFG function, with the left one participating in the inhibition of irrelevant information, while the right one is regarded as a crucial hub for suppressing premature or inappropriate responses [36, 41]. Thus, an explanation could be that the excessive attention to pain stimuli and insufficient inhibition of inappropriate responses induce patients focus on exaggerated pain, resulting in deteriorative pain cognition. Besides, we also found hypoconnectivity of left DLPFC and right SMG and hyperconnectivity of right DLPFC and left putamen were associated with poor treatment outcomes of pain vigilance. As part of cortico-striatal circuits, the putamen is responsible for governing the processes of reward learning and motivation, which are frequently disrupted or undermined in depressed states [42, 43]. According to the Motivation-Decision pain theory, perceived pain can be defined by subtracting simultaneous reward from the actual pain [44]. Hence, collaborating with DLPFC (emotional attribution), subjective pain perception is increased owing to the diminished reward experiences and increased internal attention, further increasing the fear and vigilance of pain.

Despite decades studies documented the vital role of pain in the development of depression, our study was the first one to investigate the functional circuit of DLPFC and the modulatory impact of TMS in adolescent depression with a focus on pain-related cognitive processing. However, several limitations should be addressed. First, we identified the abnormal FC patterns by case-control study. Characterizing brain contributions to individual variability in pain processing and cognition would provide stronger evidence on the personalized pain medicine, future studies could focus on the personalized target identified by individual level study. Second, the non-blinding of patients and lack of sham control may increase the risk for a Type I error in our study. Although our data demonstrated significant efficacy of add-on DLPFC-TMS in the pain cognition, these findings need to be further validated in double-blind randomized sham-controlled trial with a large sample size. Moreover, given the heterogeneity of depression and multiple dimensions of pain, a more comprehensive description of data, including clinical characteristics (e.g., age of onset, duration) and pain components (e.g., pain sensitivity and pain anticipation), would improve our understanding of pain in adolescents with depression.

## Conclusion

Together, negative cognitive response evoked by painful stimuli in adolescents with depression may be due to altered interactions between DLPFC and a set of brain regions of impulse controlling, attention, self-referencing, and reward factors. DLPFC can act as a connection linking cognitive control and pain processing, stimulation over this region could improve the pain cognition and the FC patterns of DLPFC could predict such efficacy.

## Supplementary Information

Supplementary Material 1.

## Data Availability

The datasets generated and analysed during the current study are not publicly available due to privacy and ethical restrictions, but are available from the corresponding author on reasonable request.

## Abbreviations

DLPFC: Dorsolateral prefrontal cortex
TMS: Transcranial magnetic stimulation
FC: Functional connectivity
PCS: Pain Catastrophizing Scale
PVAQ: Pain Vigilance and Awareness Questionnaire
IFG: Inferior frontal gyrus
SMG: Supramarginal gyrus
HAMD-24: 24 Item Hamilton Depression Rating Scale
HAMA: Hamilton Anxiety Rating Scale
BOLD: Blood Oxygenation Level Dependent
ROI: Region of interest
RMT: Resting motor threshold
HF: High-frequency
LF: Low-frequency
CI: Confidence intervals
