# Supplementary material for: Exploring the connectivity of dorsolateral prefrontal cortex and the modulatory impact of transcranial magnetic stimulation in adolescents with depression: a focus on pain-related cognitive processing
Source: BMC Psychiatry. 2024 Nov 27;24:852. doi: 10.1186/s12888-024-06321-x (PMC11600770; doi:10.1186/s12888-024-06321-x)
Supplement: Supplementary file 1 — Supplementary Material 1. [file 12888_2024_6321_MOESM1_ESM.docx]

**Table S1 Demographics and clinical characteristics for responders and non-responders in Add-on TMS group.**

|  | Responders  (N=13) | Non-responders (N=17) | *t* or *χ²* | *P* value |
| --- | --- | --- | --- | --- |
| Age, mean (SD) | 16.23 (2.006) | 15.71 (1.993) | 0.713 | .482 |
| Gender (male/female) | 3/10 | 2/15 | 0.679 | .410 |
| Education level, mean (SD) | 9.69 (2.658) | 9.71 (1.993) | -0.016 | .987 |
| HAMD-24 score, mean (SD) | 21.23 (4.456) | 25.06 (6.600) | -1.798 | .083 |
| HAMA score, mean (SD) | 13.77 (4.381) | 18.41 (4.273) | -2.917 | .007 |

TMS, Transcranial Magnetic Stimulation. HAMD-24, 24 item Hamilton Depression Rating Scale. HAMA, Hamilton Anxiety Rating Scale.

**Table S2 Changes of the pain cognition** **in responders and non-responders in Add-on TMS group.**

| Measure | Responders  (N=13) | Non-responders (N=17) | Difference(95%CI) | *P* value^a^ | Cohen’s *d* |
| --- | --- | --- | --- | --- | --- |
|  |  |  |  |  |  |
| **PVAQ** **score**, mean (SD) | | | | | |
| Baseline | 34.54 (12.177) | 36.47 (12.099) | -1.932 (-11.089, 7.224) | .669 | -0.15 |
| Post-intervention | 21.31 (11.123) | 29.59 (13.167) | -8.281 (-17.588, 1.027) | .079 | -0.65 |
| Difference(95%CI) | 13.231 (6.072, 20.390) | 6.882 (-0.641, 14.406) | N/A | N/A | N/A |
| *P* value^b^ | .002 | .070 | N/A | N/A | N/A |
| Cohen’s *d* | 1.13 | 0.54 | N/A | N/A | N/A |
| **PCS** **total score**, mean (SD) | | | | | |
| Baseline | 18.69 (10.467) | 27.24 (11.579) | -8.543 (-16.932, -0.154) | .046 | -0.77 |
| Post-intervention | 11.62 (9.605) | 19.18 (10.082) | -7.561 (-15.018, -0.104) | .047 | -0.77 |
| Difference(95%CI) | 7.077 (2.341, 11.812) | 8.059 (3.687, 12.431) | N/A | N/A | N/A |
| *P* value | .007 | .001 | N/A | N/A | N/A |
| Cohen’s *d* | 0.70 | 0.74 | N/A | N/A | N/A |
| **PCS-rumination sub-score**, mean (SD) | | | | | |
| Baseline | 5.54 (3.971) | 7.88 (3.604) | -2.344 (-5.186, 0.498) | .102 | -0.62 |
| Post-intervention | 3.69 (3.301) | 5.12 (2.998) | -1.425 (-3.879, 0.938) | .227 | -0.45 |
| Difference(95%CI) | 1.846 (0.269, 3.423) | 2.765 (1.154, 4.376) | N/A | N/A | N/A |
| *P* value | .025 | .002 | N/A | N/A | N/A |
| Cohen’s *d* | 0.51 | 0.83 | N/A | N/A | N/A |
| **PCS-helplessness sub-score**, mean (SD) | | | | | |
| Baseline | 8.77 (5.036) | 12.53 (6.276) | -3.760 (-8.120, 0.600) | .088 | -0.66 |
| Post-intervention | 5.08 (4.699) | 9.00 (5.906) | -3.923 (-7.890, 0.044) | .060 | -0.73 |
| Difference(95%CI) | 3.692 (1.347, 6.038) | 3.529 (0.730, 6.329) | N/A | N/A | N/A |
| *P* value | .005 | .017 | N/A | N/A | N/A |
| Cohen’s *d* | 0.76 | 0.58 | N/A | N/A | N/A |
| **PCS-****magnification sub-score**, mean (SD) | | | | | |
| Baseline | 4.38 (2.434) | 6.82 (2.604) | -2.439 (-4.350, -0.528) | .014 | -0.97 |
| Post-intervention | 2.85 (2.478) | 5.06(2.727) | -2.213 (-4.192, -0.233) | .030 | -0.85 |
| Difference(95%CI) | 1.538 (0.151, 2.926) | 1.765 (0.744, 2.785) | N/A | N/A | N/A |
| *P* value | .033 | .002 | N/A | N/A | N/A |
| Cohen’s *d* | 0.62 | 0.66 | N/A | N/A | N/A |

^a^*P* value of independent-samples *t* test. ^b^ *P* value of paired *t* test. TMS, Transcranial Magnetic Stimulation. PVAQ, Pain Vigilance and Awareness Questionnaire. PCS, Pain Catastrophizing Scale. N/A: not applicable.
